# Effect of Intrahepatic Cholestasis of Pregnancy on Neonatal Birth Weight: A Meta-Analysis

**DOI:** 10.4274/jcrpe.4930

**Published:** 2018-02-26

**Authors:** Li Li, Yuan-Hua Chen, Yuan-Yuan Yang, Lin Cong

**Affiliations:** 1 First Affiliated Hospital of Anhui Medical University, Department of Obstetrics and Gynecology, Hefei, China; 2 Anhui Medical University, Department of Histology and Embryology, Hefei, China

**Keywords:** Intrahepatic cholestasis, pregnancy, birth weight, meta-analysis

## Abstract

**Objective:**

To evaluate the effect of intrahepatic cholestasis of pregnancy (ICP) on neonatal birth weight.

**Methods:**

Potential articles were identified by searching PubMed and Web of Science databases on April 30^th^, 2017. Using the Mantel-Haenszel random-effects or fixed-effects model, outcomes were summarized through weighted mean difference (WMD) and 95% confidence intervals (CI). Potential publication bias was tested using a funnel plot and the methods of Egger’s regression and Begg’s test.

**Results:**

A total of eight studies were included in our meta-analysis. Six studies reported data on neonatal birth weight in ICP and control pregnancies. Pooled data from the six studies showed that the birth weight in the ICP group was significantly lighter than in the control group. The overall pooled WMD was -175 g (95% CI: -301, -48). Meanwhile, pooled data from the other two studies indicated that the birth weight in the late-onset ICP group was heavier than in the early-onset ICP group (WMD: 267 g, 95% CI: 168, 366).

**Conclusion:**

Neonatal birth weights in ICP pregnancies were lower than in normal pregnancies. Furthermore, early-onset ICP is associated with a lower birth weight than late-onset ICP.

## What is already known on this topic?

Several studies have demonstrated that intrahepatic cholestasis of pregnancy is associated with fetal growth, but the results are inconsistent.

## 

### What this study adds?

Neonatal birth weights of intrahepatic cholestasis of pregnancy infants were lower than that of normal pregnancies. Furthermore, early-onset intrahepatic cholestasis of pregnancy is associated with a lower birth weight than late-onset intrahepatic cholestasis of pregnancy.

## Introduction

Intrahepatic cholestasis of pregnancy (ICP) is a pregnancy-specific liver disease that usually occurs during the late second or third trimesters of pregnancy. The clinical characteristics of ICP are unexplained maternal pruritus, altered liver function and increased fasting serum bile acids (>10 mmol/L) in previously healthy pregnant women (1,2). There are differences in its prevalence in different regions and countries. It occurs in approximately 0.1% to 1.5% of pregnancies in Europe and the United States (3,4), while its prevalence ranges from 11.8% to 27.6% in Chile and Bolivia, varying by ethnic origin (5,6). Currently, the etiology of this condition is not fully understood and it is estimated that racial, genetic, hormonal, nutritional and environmental factors play a role (7). Although ICP is a benign disease, it can lead to increased fetal morbidity and mortality, particularly with regard to neonatal respiratory Distress syndrome, preterm delivery, fetal distress and sudden intrauterine fetal death (8). Several studies have demonstrated an association between ICP and fetal growth, but the results are inconsistent. A large, population-based cohort study reported a significant increase in the incidence of large for gestational age (LGA) infants in pregnancies complicated by ICP even after controlling for diabetes and preeclampsia (9). Martineau et al (10) also reported that ICP was associated with increased fetal growth. However, a study from Turkey found that ICP may lead to low birth weight (11) and a similar result was reported in another study (12). To further investigate the possible association between ICP and neonatal birth weight, we conducted this meta-analysis to summarize all available evidence.

## Methods

### Search Strategy and Selection Criteria

Relevant literature published before April 30^th^, 2017 was identified by searching PubMed and Web of Science databases. The search strategy was based on the following keywords: “cholestasis”, “intrahepatic cholestasis”, “pregnancy”, “pregnant”, “birth weight”, “birthweight”, “fetal growth restriction” and “intrauterine growth restriction”. Only publications in English or Chinese were included. Relevant eligible literatures were also scanned through cross-references of identification in the reference lists within both original and review articles. In situations where key information relevant to the meta-analysis was missing, the authors were contacted to supply additional data. We employed EndNote for managing bibliographies and references. An essential feature of EndNote is that it allowed us to identify duplicates of studies found through different, overlapping databases. Studies were included in the analysis if the sample included: a) patients diagnosed with ICP; and b) birth weight measurements and if birth weight was measured as a continuous variable. If more than one study was identified for the same population, the more recent study or the one providing more information was selected. Studies were excluded if they were reported as case series, letters, review articles or editorials, and did not meet the above criteria. All analyses were based on previous published studies, thus no ethical approval and patient consent are required.

### Data Extraction

After initial evaluation, two reviewers (L.L. and C.Y.H.) independently and carefully evaluated the articles and performed the data extraction according to the selection criteria. The following variables in each study were extracted: first author, year of publication, study country, age, gestational age at delivery, number of pregnancies (ICP and control; early-onset ICP and late-onset ICP), definition of ICP. When discrepancies existed, the case was discussed with another reviewer (Y.Y.Y.) until a consensus was reached.

### Quality Assessment

The methodological quality of each study was independently assessed by two reviewers (L.L. and C.Y.H.) using the Newcastle-Ottawa quality assessment scale (13). Ten questions were assessed and each satisfactory answer received one point, resulting in a maximum score of nine. When publications had scores of ≥6, they were graded as high-quality. When there was a disagreement, it was solved by consensus of the whole team.

### Statistical Analysis

All statistical analyses were carried out with the Stata 12.0 program (Stata-Corp, College Station, TX USA). Weighted mean difference (WMD) can be used as a summary statistic in meta-analysis when outcome measurements in all studies are made on the same scale. So using the Mantel-Haenszel random-effects or fixed-effects model, outcomes were summarized through WMD and 95% confidence intervals (CI). Statistical heterogeneity was measured using the chi-square test on Q statistic, which was quantified by I-squared values, assuming that I-squared values of 25, 50 and 75% were nominally assigned as low, moderate and high estimates, respectively (14). P<0.10 or I-squared >50% indicates that heterogeneity existed among the studies, so a random-effects model (Mantel-Haenszel method) should be used. In order to assess the impact on the results of a single study, we conducted a sensitivity analysis of each study by excluding each study one by one and recalculating the combined estimates on remaining studies. Potential publication bias was tested using the funnel plot and the method of Egger’s regression and Begg’s test. P≤0.05 indicated the presence of statistically significant findings.

## Results

**Figure 1:**
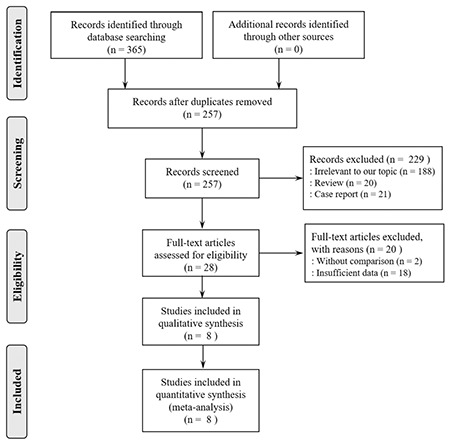
Flow diagram of the study selection process

The initial literature search revealed 365 relevant articles on the association of ICP and neonatal birth weight. After the careful screening process, 108 studies were excluded as they were duplicates. Two hundred twenty-nine studies were rejected because 188 of these reports were irrelevant to our topic, 20 were review articles and 21 were case reports. The remaining 28 relevant studies were selected for detailed evaluation. Of these, 20 publications did not meet the inclusion criteria and were excluded. Finally, a total of eight studies (10,11,12,15,16,17,18,19) were included in our meta-analysis. Figure 1 outlines the literature review and study selection process. The characteristic of each article included in this meta-analysis is shown in Table 1. Two of these studies were performed in Turkey (11,16), two in China (12,19), one in Finland (18), one in the United Kingdom (10), one in USA (15) and one in Poland (17). Six of the studies (10,12,15,16,17,18) were conducted to explore the effect of ICP on birth weight (control vs ICP), and two studies (11,19) were conducted to compare early-onset (<32 weeks gestation) and late-onset (≥32 weeks gestation) ICP pregnancies. The quality of study was assessed by Newcastle-Ottawa quality assessment scale. The quality scores ranged from six to eight and showed that the studies were of acceptable quality.

### Meta-Analysis Results

**Figure 2:**
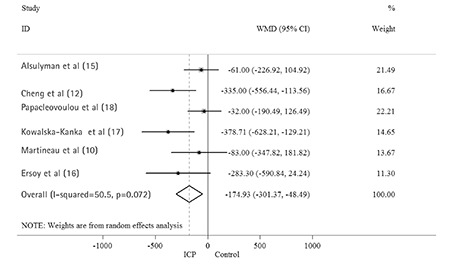
Forest plot of pooled estimated weighted mean difference with 95% confidence interval of birth weight between intrahepatic cholestasis of pregnancy and normal pregnancies. All studies were sorted by publication year CI: confidence interval, ICP: intrahepatic cholestasis of pregnancy, WMD: weighted mean difference, ID: infectious disease

**Figure 3:**
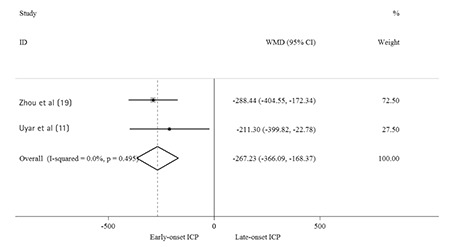
Forest plot of pooled estimated weighted mean difference with 95% confidence interval of birth weight between early-onset and late-onset intrahepatic cholestasis of pregnancy pregnancies. All studies were sorted by publication year. ID: infectious disease, WMD: weighted mean difference, ICP: intrahepatic cholestasis of pregnancy

A total of six studies reported data on neonatal birth weight in ICP and control pregnancies. Pooled data from all the six studies showed that the birth weight in the ICP group was significantly lighter than those in the control group. The overall pooled WMD was -175 g (95% CI: -301, -48). The I-squared statistic (I-squared=50.5%, p=0.072) indicated moderate heterogeneity (Figure 2). Two studies reported data on neonatal birth weight in early-onset (<32 weeks) and late-onset (≥32 weeks) ICP pregnancies. Combined data from these two studies indicated a significant difference between the groups (Figure 3). The birth weight in the late-onset ICP group was heavier than that in the early-onset ICP group (WMD: 267 g, 95% CI: 168, 366). There was low heterogeneity (I-squared=0.0%, p=0.495).

### Sensitivity Analysis

**Figure 4:**
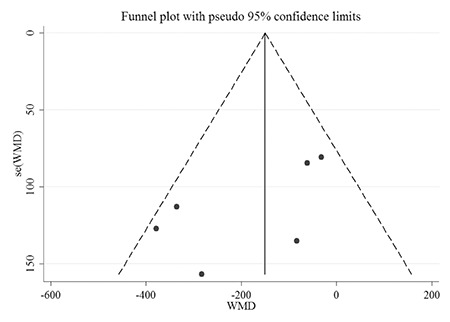
Funnel plot of the 6 studies included in the meta-analysis WMD: weighted mean difference

To confirm the stability and reliability of the meta-analysis, sensitivity analysis was performed by repeating the calculation of pooled WMD (95% CI) when any single study was deleted. Figure 4 showed that the corresponding pooled WMD (95% CI) ranged from -215 (-355, -75) g to -135 (-255, -15) g and was not substantially altered. The confidence limits of the overall estimate are -301 and -48 and -361 and -11 are the most extreme confidence limits of the estimates, calculated when any one study was omitted. The statistically similar results indicated that no single study had any influence on the stability of the overall WMD estimate in this meta-analysis.

### Publication Bias

**Figure 5:**
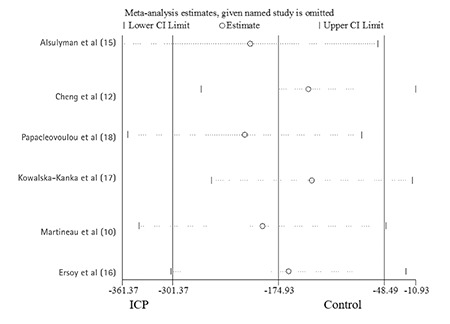
Sensitivity analysis for individual studies on the summary effect. All studies were sorted by publication year ICP: intrahepatic cholestasis of pregnancy, CI: confidence interval

The graphical funnel plots appeared to be symmetrical (Figure 5), and the Begg’s test (z=1.13, p=0.260) and Egger’s test (t=-1.93, p=0.126) indicated there was no strong evidence for publication bias.

## Discussion

This systematic review and meta-analysis was conducted to assess the effect of ICP on neonatal birth weight. The data showed that birth weight in the ICP group was significantly lighter than that in the control group (WMD: -175g, 95% CI: -301, -48). In addition, birth weight was significantly higher in late-onset compared with early-onset ICP cases. Although ICP is a relatively nonthreatening condition to mothers, there are serious risks for the fetus. It is linked with a higher risk of fetal death, meconium staining of amniotic fluid, fetal distress and preterm delivery (20,21,22). Fetal growth *in utero* is a complex process and involves interactions between mother, fetus and placenta. Maternal and fetal endocrine status, genetic predisposition and available substrates have an impact on fetal growth and also determine birth weight (23). Several studies have demonstrated that ICP has an influence on fetal growth. However, there was a wide variation in the results reported in the conducted studies (10,12,17,24,25). In a study from Poland investigating 73 pregnant women, it was reported that the babies of ICP mothers had a lower birth weight (17). These findings are consistent with a study from China (12). In contrast, a retrospective case-control study reported increasing customized, singleton, birth-weight centiles with advancing gestational age in cholestatic pregnancies (24). In another study, the incidence of LGA infants of ICP mothers was higher, compared with the incidence of SGA infants (25). Some studies have also focused on the association between ICP cases of different gestational onset time and birth weight. In a retrospective analysis (26), it was reported that early-onset ICP is associated with a higher frequency of adverse fetal outcomes than late-onset ICP, especially in severe disease. In this systematic review and meta-analysis, neonatal birth weights were found to be lower in early-onset ICP than late-onset ICP, a finding which indicates that early-onset ICP has greater influence on birth weight. In our meta-analysis, there is no potential risk of publication bias. When we excluded one study per iteration, the range of variation of the overall result is also smaller, which suggests that no one study can significantly alter the findings. Furthermore, the overall quality was acceptable in all of the studies included. However, there are still some limitations. Firstly, some included studies were conducted using medical databases, raising the possibility of coding inaccuracy. Secondly, a heterogeneity between studies was observed in the study. In addition, the results relied on aggregated published data. In the future, large-scale prospective studies will possibly provide a more accurate association between ICP and birth weight.

### Study Limitations

There are still some limitations. First, some included studies were conducted using medical databases, raising the possibility of coding inaccuracy. Second, a heterogeneity between studies was observed in the study. In addition, the results relied on aggregated published data. In in the future, large-scale prospective studies will possibly provide a more accurate association between ICP and birth weight.

## Conclusion

In summary, this meta-analysis demonstrated that neonatal birth weight is lower in ICP pregnancies than in normal pregnancies. Furthermore, early-onset ICP is associated with a lower birth weight than late-onset ICP. 

## Figures and Tables

**Table 1:**
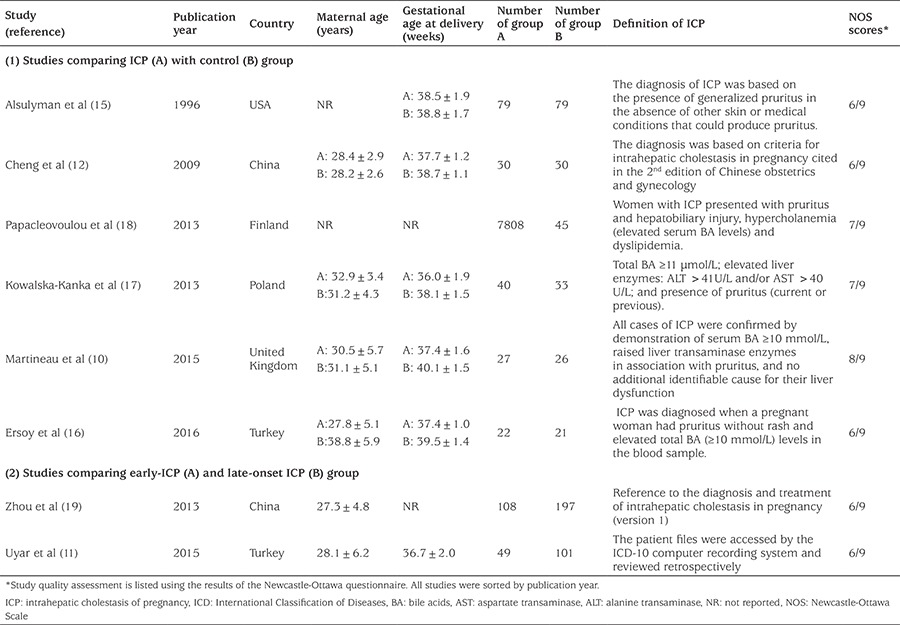
Characteristics of the studies included in the meta-analysis

## References

[1] Estiu MC, Monte MJ, Rivas L, Moiron M, Gomez-Rodriguez L, Rodriguez-Bravo T, Marin JJ, Macias RI (2015). Effect of ursodeoxycholic acid treatment on the altered progesterone and bile acid homeostasis in the mother-placenta-foetus trio during cholestasis of pregnancy. Br J Clin Pharmacol.

[2] Lo JO, Shaffer BL, Allen AJ, Little SE, Cheng YW, Caughey AB (2015). Intrahepatic cholestasis of pregnancy and timing of delivery. Journal of Maternal-Fetal & Neonatal Medicine.

[3] Joshi D, James A, Quaglia A, Westbrook RH, Heneghan MA (2010). Liver disease in pregnancy. Lancet.

[4] Williamson C, Geenes V (2014). Intrahepatic cholestasis of pregnancy. Obstet Gynecol.

[5] Reyes H, Taboada G, Ribalta J (1979). Prevalence of intrahepatic cholestasis of pregnancy in La Paz, Bolivia. J Chronic Dis.

[6] Reyes H, Gonzalez MC, Ribalta J, Aburto H, Matus C, Schramm G, Katz R, Medina E (1978). Prevalence of intrahepatic cholestasis of pregnancy in Chile. Ann Intern Med.

[7] Diken Z, Usta IM, Nassar AH (2014). A clinical approach to intrahepatic cholestasis of pregnancy. Am J Perinatol.

[8] Saleh MM, Abdo KR (2007). Intrahepatic cholestasis of pregnancy: review of the literature and evaluation of current evidence. J Womens Health (Larchmt).

[9] Wikstrom Shemer E, Marschall HU, Ludvigsson JF, Stephansson O (2013). Intrahepatic cholestasis of pregnancy and associated adverse pregnancy and fetal outcomes: a 12-year population-based cohort study. BJOG.

[10] Martineau MG, Raker C, Dixon PH, Chambers J, Machirori M, King NM, Hooks ML, Manoharan R, Chen K, Powrie R, Williamson C (2015). The metabolic profile of intrahepatic cholestasis of pregnancy is associated with impaired glucose tolerance, dyslipidemia, and increased fetal growth. Diabetes Care.

[11] Uyar I, Gulhan I, Oztekin D, Gezer C, Ekin A, Karaca Kurtulmus S, Ozeren M (2015). Intrahepatic cholestasis of pregnancy may lead to low birth weight. Turk J Med Sci.

[12] Cheng XY, Zhang LJ, Lin L, Liu J, Ding YL (2009). Relationship of fetal total bile acid and the change of fetal pancreas endocrine secretion and its impact on fetal growth and development in intrahepatic cholestasis of pregnancy. Zhonghua Fu Chan Ke Za Zhi.

[13] Stang A (2010). Critical evaluation of the Newcastle-Ottawa scale for the assessment of the quality of nonrandomized studies in meta-analyses. Eur J Epidemiol.

[14] Fan D, Xia Q, Liu L, Wu S, Tian G, Wang W, Wu S, Guo X, Liu Z (2017). The Incidence of Postpartum Hemorrhage in Pregnant Women with Placenta Previa: A Systematic Review and Meta-Analysis. PLoS One.

[15] Alsulyman OM, Ouzounian JG, Ames-Castro M, Goodwin TM (1996). Intrahepatic cholestasis of pregnancy: perinatal outcome associated with expectant management. Am J Obstet Gynecol.

[16] Ersoy AO, Kirbas A, Ozler S, Ersoy E, Ozgu-Erdinc AS, Ergin M, Erkaya S, Uygur D, Danisman N (2016). Maternal and fetal serum levels of caspase-cleaved fragments of cytokeratin-18 in intrahepatic cholestasis of pregnancy. J Matern Fetal Neonatal Med.

[17] Kowalska-Kanka A, Maciejewski T, Niemiec KT (2013). The concentrations of bile acids and erythropoietin in pregnant women with intrahepatic cholestasis and the state of the fetus and newborn. Med Wieku Rozwoj.

[18] Papacleovoulou G, Abu-Hayyeh S, Nikolopoulou E, Briz O, Owen BM, Nikolova V, Ovadia C, Huang X, Vaarasmaki M, Baumann M, Jansen E, Albrecht C, Jarvelin M-R, Marin JJG, Knisely AS, Williamson C (2013). Maternal cholestasis during pregnancy programs metabolic disease in offspring. Journal of Clinical Investigation.

[19] Zhou L, Qi HB, Luo X (2013). [Analysis of clinical characteristics and perinatal outcome of early-onset intrahepatic cholestasis of pregnancy]. Zhonghua Fu Chan Ke Za Zhi.

[20] Brouwers L, Koster MP, Page-Christiaens GC, Kemperman H, Boon J, Evers IM, Bogte A, Oudijk MA (2015). Intrahepatic cholestasis of pregnancy: maternal and fetal outcomes associated with elevated bile acid levels. Am J Obstet Gynecol.

[21] Rook M, Vargas J, Caughey A, Bacchetti P, Rosenthal P, Bull L (2012). Fetal outcomes in pregnancies complicated by intrahepatic cholestasis of pregnancy in a Northern California cohort. PLoS One.

[22] Kondrackiene J, Beuers U, Zalinkevicius R, Tauschel HD, Gintautas V, Kupcinskas L (2007). Predictors of premature delivery in patients with intrahepatic cholestasis of pregnancy. World J Gastroenterol.

[23] Wang HQ, Lai HL, Li Y, Liu QF, Hu S, Li L (2016). The Relationship between Maternal Gestational Impaired Glucose Tolerance and Risk of Large-for-Gestational-Age Infant: A Meta-Analysis of 14 Studies. J Clin Res Pediatr Endocrinol.

[24] Martineau M, Raker C, Powrie R, Williamson C (2014). Intrahepatic cholestasis of pregnancy is associated with an increased risk of gestational diabetes. Eur J Obstet Gynecol Reprod Biol.

[25] Baliutaviciene D, Zubruviene N, Zalinkevicius R (2011). Pregnancy outcome in cases of intrahepatic cholestasis of pregnancy. Int J Gynaecol Obstet.

[26] Jin J, Pan SL, Huang LP, Yu YH, Zhong M, Zhang GW (2015). Risk factors for adverse fetal outcomes among women with early- versus late-onset intrahepatic cholestasis of pregnancy. Int J Gynaecol Obstet.

